# Retraction: Lithium chloride inhibits titanium particle-induced osteoclastogenesis by inhibiting the NF-κB pathway

**DOI:** 10.18632/oncotarget.28684

**Published:** 2025-01-21

**Authors:** Xuanyang Hu, Zhirong Wang, Jiawei Shi, Xiaobin Guo, Liangliang Wang, Zichuan Ping, Yunxia Tao, Huilin Yang, Jun Zhou, Yaozeng Xu, Dechun Geng

**Affiliations:** ^1^Department of Orthopedics, The First Affiliated Hospital of Soochow University, Suzhou, China; ^2^Department of Orthopedics, Zhang Jia Gang Hospital of Traditional Chinese Medicine, Zhangjiagang, China; ^*^These authors have contributed equally to this work

**This article has been retracted:** Oncotarget has completed its investigation of this paper. We found two instances of overlap between images in this article and reuse of the images from previously published papers from the same lab. Thus, in Figure 3, panels E and G, presenting bone marrow-derived macrophages TRAP staining, contain an overlap between images which should show different experimental conditions. The same issue occurred in panel C of Figure 6. In addition, there are three common immunohistochemical images between Figure 2D and figures in two earlier published papers, Figure 6 of [1] and Figure 3 of [2], describing different types of the experiments. Corresponding author Yaozeng Xu acknowledged these duplications, stating, “We take full responsibility for this oversight and recognize that this error may have impacted the scientific accuracy of our work. Given the importance of maintaining the rigor and reliability of published research, and after discussing the matter thoroughly with my co-authors, we have come to the conclusion that retracting the manuscript is the most responsible course of action.” In light of these circumstances, the Editorial decision was made to retract this paper. All authors agreed with the decision.

Original article: Oncotarget. 2017; 8:83949–83961. 83949-83961. https://doi.org/10.18632/oncotarget.20000
